# A small-gap electrostatic micro-actuator for large deflections

**DOI:** 10.1038/ncomms10078

**Published:** 2015-12-11

**Authors:** Holger Conrad, Harald Schenk, Bert Kaiser, Sergiu Langa, Matthieu Gaudet, Klaus Schimmanz, Michael Stolz, Miriam Lenz

**Affiliations:** 1 Fraunhofer Institute for Photonic Microsystems, 01109 Dresden, Germany; 2 Chair of Micro and Nano Systems, Brandenburg University of Technology Cottbus-Senftenberg, 03013 Cottbus, Germany

## Abstract

Common quasi-static electrostatic micro actuators have significant limitations in deflection due to electrode separation and unstable drive regions. State-of-the-art electrostatic actuators achieve maximum deflections of approximately one third of the electrode separation. Large electrode separation and high driving voltages are normally required to achieve large actuator movements. Here we report on an electrostatic actuator class, fabricated in a CMOS-compatible process, which allows high deflections with small electrode separation. The concept presented makes the huge electrostatic forces within nanometre small electrode separation accessible for large deflections. Electrostatic actuations that are larger than the electrode separation were measured. An analytical theory is compared with measurement and simulation results and enables closer understanding of these actuators. The scaling behaviour discussed indicates significant future improvement on actuator deflection. The presented driving concept enables the investigation and development of novel micro systems with a high potential for improved device and system performance.

## Abstract (web summary)

Most electrostatic actuators suffer from an operational instability, the so-called pull-in effect. Here, the authors report an electrostatic actuator concept which makes the huge electrostatic forces within nanometre small electrode separation accessible for large deflections.

## Graphical abstract

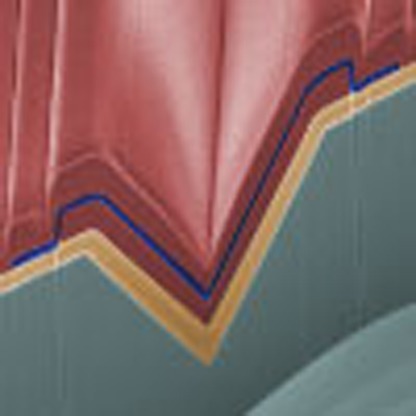

Electrostatic actuation provides efficient, low-power and fast-response driving and control of movable micro and nano structures, for example, in high frequency switches, oscillators, mirrors, motors and sensors. Owing to a prominent scaling behaviour, electrostatic forces have gained a dominant position in micro and nano device actuation. Every downscaling in electrode gap further expands its superiority over other physical driving principles, such as electromagnetic or piezoelectric actuation. In addition, it is at least as important that electrostatic actuators are easy to fabricate with standard micro and nanotechnologies, and that they offer the advantage of integration with complementary metal-oxide-semiconductor (CMOS).

However, most electrostatic actuators suffer from an operational instability – the so-called pull-in effect – which was first discovered on resonant gate transistors in 1967 (ref. 1). The highly non-linear dependence of the electrostatic force on the separation of a mechanically suspended electrode to a fixed electrode results in a prevailing electrostatic force once a critical deflection is reached. From this point on, the mechanical restoring force is always smaller than the electrostatic attraction, which leads to snapping and subsequently sticking of the electrodes. Theory suggests a characteristic travel range of typically 1/3 of the electrode gap for the static^1^,^2^, 1/2 for the dynamic^2^ and 
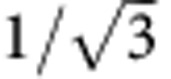
 for the resonant case^3^. In practice, the pull-in effect causes significantly higher constraints as fabrication tolerances and safety margins need to be considered. Pull-in related effects, such as stiction, adhesion, electrical discharge and dielectric charging, are considered to be the primary causes of device failure for a large variety of electrostatically actuated micro devices^4^. The main challenges to the future development of electrostatic micro and nano actuators and sensors are to control the pull-in instability and to extend the travel range beyond the pull-in limit.

A minority of electrostatic actuator classes, such as in-plane actuated comb drives^5^ and resonant out-of-plane moving comb drives^6^, enable travel magnitudes that are significantly larger than the electrode separation. Pull-in instabilities do not affect the fundamental operation principle but needs to be considered as parasitic effects on these concepts. Typical electrode separations are in the lower micrometre range, but further significant downscaling is rather challenging due to the fabrication process. Recent publications have reported various attempts to enhance the controllable travelling range of static or quasi-static out-of-plane operating electrostatic actuators^4^. Among these are electrical control strategies^7^, leverage methods^8^, active or passive suspension spring stiffening methods^9^,^10^, mechanical non-linearities within the actuated micro component^11^,^12^, a non-constant electrode separation^13^,^14^ and the usage of electrostatic fringing fields^15^. Thus far, travel ranges up to 94 % of initial electrode separation have been reported^13^.

We believe that an extension of the travel range beyond the pull-in limit can be efficiently provided by a specific leverage approach. In the approach we present here, the high electrostatic forces generated by small electrode gaps are transformed into high deflection magnitudes. Some of the best-known mechanical levers are bimorph actuators^16^, where small lateral thermomechanical, piezoelectric or other electroactive material strain results in high deflection magnitudes. Combining electrostatic forces with the bimorph leverage principle, lead to the development of a novel class of efficient, CMOS integrable electrostatic actuators. In the concept presented, electrostatic forces are transformed into lateral forces via non-planar electrode geometries. This induces a lateral strain at the surface of a cantilever and forces the cantilever to bend. In other words, electrostatic forces stretch or compress the surface of a cantilever and cause the cantilever deflection. With this novel electrostatic actuator concept, the cantilever tip deflection depends on electrostatic forces instead of the electrostatically generated electrode deflection. This enables reasonable actuator deflection to be achieved with very small electrode separation, and enables travel ranges widely beyond the pull-in limit. The small electrode separation can keep the control voltage at moderate levels, and downscaling the electrode separation can further reduce the control voltage. The concept proposed of a novel electrostatic actuator class operates with electrode gaps far below micrometre, hence will now be referred to as nano electrostatic drive (NED).

In this work, we present the theoretical description, a fabrication process and first characterization results of NED actuators. The measured deflection curves are fitting well with the prediction done by analytical and simulation models. We demonstrate a travel range of 136 % on characterized samples of these cantilevered electrostatic actuators. The scaling of NED principle discussed provides the information on how to improve the presented actuation concept and achieve significant higher travel ranges.

## Results

### Theory of the novel electrostatic actuators

**Figure 1:**
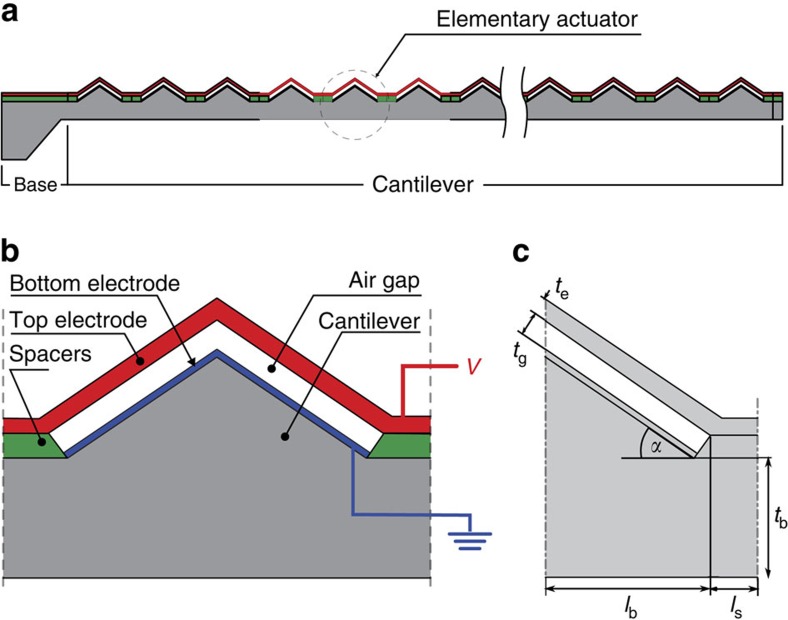
Schematic cross-section of a Λ-shaped NED. (**a**) Schematic design of a whole electrostatically actuated cantilever. (**b**) Schematic image of a single elementary actuator cell with the indication of ground (blue) and control potential (red). (**c**) Illustration of a half elementary actuator cell and the fundamental geometric parameters.

The NED actuator is composed of periodically repeated electrostatic-force-based elementary actuator cells processed at the surface of a cantilever. Figure 1 illustrates schematic cross-sections of the whole actuator (Fig. 1a), the elementary actuator cell (Fig. 1b) and a half elementary actuator cell labelled with the fundamental parameters (Fig. 1c). The role of the elementary actuator cells is to transform the electrostatic forces into lateral mechanical forces, which consequently leads to a bending of the cantilever. Each elementary actuator cell is a section part of the cantilever (Fig. 1a). A single actuator cell is composed of a base on which two electrodes are placed, separated by two spacers on the left and right-hand side of an air gap. The difference of electrostatic potential applied between the top and the bottom electrode generates an electrostatic force that creates a bending of the top electrode, in addition to its downward displacement. The spacers constrain the top electrode from large deflection by connecting it with other parts of the structure, but it produces significant strain in the upper electrode. The top electrode transforms this surface strain to the structure, which will consequently deform the whole actuator and lead to the bending of the associated section of the cantilever. Investigating the elementary actuator cell topography allows one to understand the transformation of electrostatic forces into cantilever bending as well as the direction of cantilever deflection. To facilitate this, we differentiate two simple cases in top electrode topography, namely V- and Λ-shaped NED actuators. The V-shaped elementary actuator cells utilize top electrode topographies that are inclined to each other and allow an upward bending of the NED actuator. The top electrode slope angle *α* is defined as a negative value in this case (Supplementary Fig. 1). Alternatively, a positive value of the slope angle *α* results in a ‘pitched roof' shaped top electrode topography (Fig. 1c). This geometry is specified as Λ-shaped elementary actuator cells, and allows the NED actuator to bend in the opposite direction.

**Figure 2:**
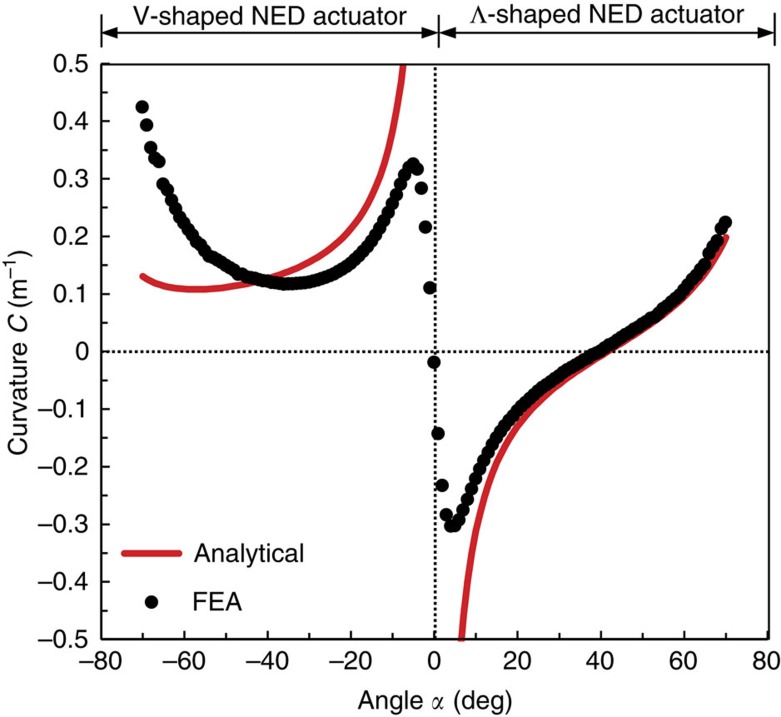
The controlled change in V- and Λ-shaped NED actuator curvature. The change in cantilever curvature as a function of the elementary actuator roof angle *α*. Results are given for the calculation by the analytical model (red line), presented in the Supplementary Information, and for the simulation using electrostatic mechanical coupled-field FEA (black dots). The electrostatic control voltage *V* of 10 V was applied across an air gap *t*_g_ of 100 nm (*ɛ*_r_=1), with elementary actuator cell length of 6 μm (*l*_b_=2.5 μm and *l*_s_=0.5 μm), a top electrode thickness *t*_e_ of 200 nm and a cantilever thickness *t*_b_ of 6 μm. The top electrodes and cantilever are assumed to be made of silicon with a modulus of elasticity *E* of 169 GPa. The spacers were assumed to be made of alumina with a modulus of elasticity *E* of 266 GPa.

Because of the elementary actuator cell periodicity (Fig. 1a), the whole cantilever has to be cylindrically deformed. Therefore, the NED cantilever deformation can be described with a curvature *C* according to state-of-the-art bimorph actuators^16^. An analytical model, presented in the Supplementary Information, allows the calculation of the curvature *C* of the cantilever, composed of V- and Λ-shaped elementary actuator cell topography, respectively. The aim of this analytical model is to understand the general behaviour of NED actuators for positive and negative angles of the V- and Λ-shaped electrode roof. The model relates the transformation of electrostatic forces into lateral mechanical forces and their action on the cantilever. It assumes a perfectly stiff top electrode and consequently neglects the displacement associated with top electrode bending. Using the analytical model, the voltage-controlled change in cantilever curvature was calculated for a representative set of geometrical parameters and is presented in Fig. 2 as a function of the roof angle *α*. In this example, the electrostatic control voltage *V* of 10 V was applied across an electrode separation of 100 nm. To validate the analytical model, a comparison with an electrostatic structural coupled finite element analysis (FEA, see Methods section) was performed. The results of the FEA simulation are presented alongside the analytical curves in Fig. 2. In both cases, the curvature will be positive for negative roof angles, with a minimum reached at −55° for the analytical model and −35° for the FEA simulation. For positive roof angles, the curvature *C* will increase from 5° to 75°, changing sign at an angle of 40°. In the FEA model, the change in top electrode deformation from a flat to a sagged surface will further shorten the distance between the spacer and the middle point of the top electrode. This extra behaviour, which is not taken into account within the analytical model, will influence the overall curvature of the cantilever for small roof angles. The sagging of the top electrode will generate additional tensile forces for roof angles between −10° and 10°. These forces will superpose the transformed forces generated by the electrostatic field. As the analytical model assumes the infinite stiffness of the top electrode, it will not consider this extra phenomenon, which results in its unrealistic predictions for angles between −10° and 10°.

**Figure 3:**
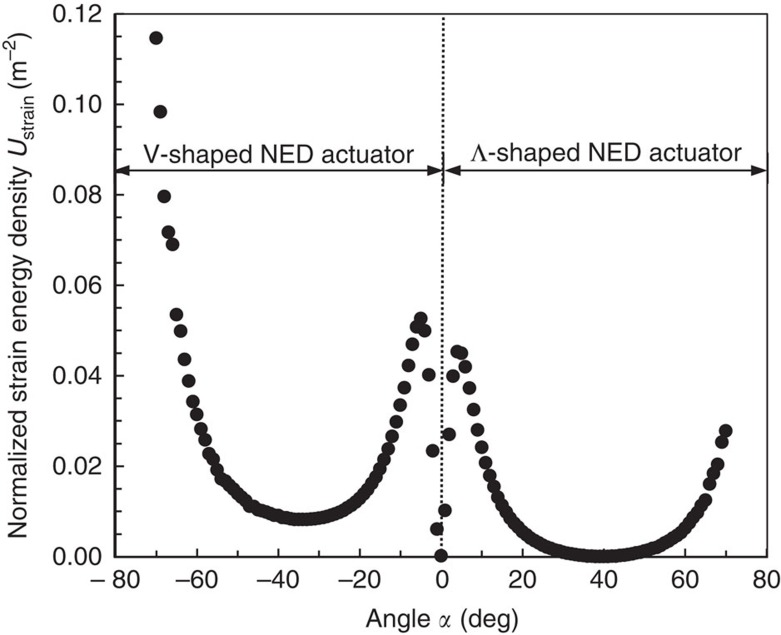
Graph presenting the strain-energy density in V- and Λ- shaped NED actuator. The strain energy as a function of the roof angle, normalized using the bending stiffness of flat roof shaped structure (*α*=0) as a reference. All FEA simulations were done for the following set of parameters: *t*_g_=100 nm, *t*_b_=6 μm, *t*_e_=200 nm, *l*_b_=2.5 μm, *l*_s_=0.5 μm, *E*=169 GPa (electrodes), *E*=266 GPa (spacer), *V*=10 V and *ɛ*_r_=1.

The strain-energy density 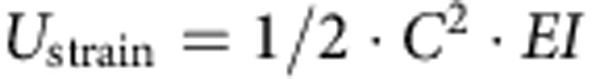 of the NED actuator, which is the strain energy per cantilever unit length, allows the evaluation of the capacity of the bending actuator to transform electrostatically generated forces into deflection. This energy depends on the active change in curvature *C* and the bending stiffness *EI* of the whole actuator. The normalized strain energy, using its normalized bending stiffness (Supplementary Fig. 2a), has been calculated by structural FEA for each roof angle (Fig. 3). The obtained curve highlights particularly large deflection capabilities for *α*≈±5° and *α*<−65°.

From the basic theory of the NED actuators, it can be understood that an active bending of cantilevers can be achieved using electrostatic forces and periodically repeated elementary actuator cells with non-planar electrode topographies. Depending on the particular arrangement of the surface topography, downward as well as upward deflectable micro actuators are possible. The topography of the elementary actuator electrode cell will influence the deflection curvature magnitude, the cantilever tip deflection and, therefore, the efficiency of the actuator. The novel NED concept was then validated with two different types of NED bending actuators fabricated with standard microelectromechanical systems (MEMS) processes.

### Sample fabrication

**Figure 4:**
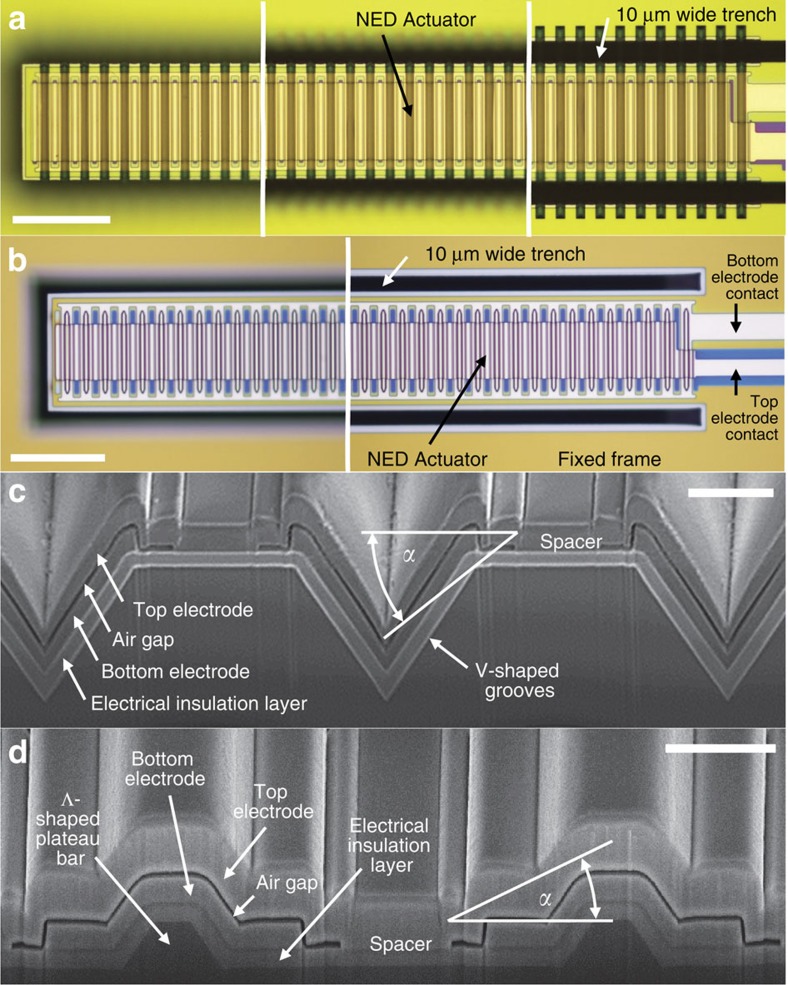
Finally released V-like and Λ-like NED actuators. (**a**) Microscope image (top view) of a released sample of V-like NED actuator at its free end, middle and clamping position (from left to right, scale bar, 50 μm). (**b**) Microscope image (top view) of a released sample of Λ-like NED actuator at its free end and clamping position (left and right, scale bar, 50 μm). (**c**) Scanning electron micrograph of a cross-section of a V-like NED actuator sample cut with a focused ion beam at the middle of the cantilever (scale bar, 2 μm). (**d**) Scanning electron micrograph of a cross-section of a Λ-like NED actuator sample cut with a focused ion beam at the middle of the cantilever (scale bar, 2 μm). Additional microscopy images are given in Supplementary Figs 8 and 9 to provide a more detailed understanding of the fabricated NED actuators.

The NED concept demands suitable top electrode topographies to transform the electrostatic forces into lateral forces. Further, particularly favourable is a small and conformal separation of two electrodes on top of a single-sided-clamped cantilever. The topography is one of the most important degrees of freedom in the optimization of the NED effect. As demonstrated in Figs 2 and 3, small structural angles in the region of −5°, +5° and angles below −65° are required for a large actuator bending and corresponding tip deflection capability. However, topography is generally an undesirable feature in semiconductor and MEMS fabrication technologies because the conformity of thin film deposition, photolithography and reactive ion-etching processes are quite sensitive to existing topography. The topography height is, therefore, limited to a few micrometres. Thus, small structural angles are favoured and the roof angle region below −65° (Figs 2 and 3) becomes less attractive. In principle, topography can be achieved with a material deposition on previously structured thin film bars or edges, with grey scale lithography^17^ and with wet isotropic or anisotropic etching on thin films or substrate materials. To validate the NED concept, we decided to utilize the common anisotropic wet etch of (100)-oriented single crystalline silicon substrate material via tetramethylammonium hydroxide (TMAH) to achieve V-shaped grooves or Λ-shaped plateau bars with {111}-oriented silicon sidewalls (Methods section and Supplementary Fig. 3). The slope angles of such manufactured sidewalls are precisely defined by an angle of 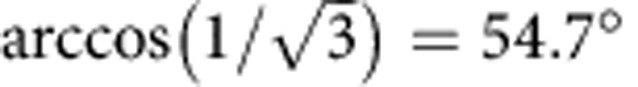 within the crystal lattice of single crystalline silicon (Supplementary Fig. 3). The essential top electrode effective structural angle *α* is defined in our concept by the lateral design of the NED elementary actuator cell, the relatively large sidewall angle of 54.7° and the depth of the V-shaped grooves or the height of the Λ-shaped plateau bars (Fig. 4). The length of the required spacers and additional lateral space needed for lithography and etching of thin films (Fig. 4) results in an absolute value of the effective structural angle *α* that is always <54.7°. Hence, only the small structural angle region between −54.7° and 54.7° is still of interest with a topography generation via TMAH etching of single crystalline silicon.

For full exploitation of the NED concept it is needed to ensure an equidistant seperation of the top and bottom electrodes. The electrostatic forces increase disproportionately with an electrode separation decrease, which means very small and conformal electrode gap sizes are of special interest. Such small electrode separation can be achieved with sacrificial layer techniques, where a thin film is removed at a final release etch. The thin film thickness will define the final electrode interspace and can be in the range of several hundred to a tenth of a nanometre with standard MEMS processes. To avoid the sticking of the electrodes due to capillary forces within wet sacrificial layer etching, supercritical drying or a vapour etchant is necessary. We fabricated test samples with 200 nm silicon dioxide thin sacrificial layer films, which were finally released etched in hydrogen fluoride vapour to create an electrode separation of ≈200 nm (Methods section and Supplementary Fig. 3). For concept verification we used amorphous titanium aluminide (TiAl) electrodes, amorphous alumina insulating material, as well as single crystalline silicon cantilever and substrate material. These materials were chosen owing to the fact that they are inert by the hydrogen fluoride vapour etchant.

With the utilized MEMS and CMOS-compatible processes (Methods section, Supplementary Fig. 3), we fabricated two different types of NED test actuators on 30-μm-thick single crystalline silicon cantilevers. Examples of finally released actuators are shown on microscopic images in Fig. 4. The fabricated actuators topographies (Fig. 4c,d) do not perfectly fit with ideal V- and Λ-shaped elementary actuator cell geometries (Fig. 1). Thus, below we named the fabricated NED actuators V-like and Λ-like NED actuators. The elementary actuator cell topography of the fabricated V-like NED actuators (Fig. 4c) was achieved by 3.5-μm-deep V-shaped grooves. With a half-cell length of 3.5 μm and because of the thin film thicknesses, the top electrode effective structural angle *α* results in −32°. Owing to a less conformal thin film deposition at topography edges and slopes, the electrode separation determined from the microscope image (Fig. 4c) varies between 226 and 110 nm. Fabricated samples of Λ-like NED actuators (Fig. 4b,d) have 1-μm-high Λ-shaped plateau bars and a half-cell length of 3.5 μm. This leads to an effective structural angle of +19°. In this case the electrode separation, as determined from the scanning electron micrograph, varies between 145 and 110 nm. The two types of fabricated NED actuator test samples work with positive and negative effective structural angles of the characteristic NED top electrode roof slope curve (Fig. 2) and were characterized regarding their quasi-static tip deflection for theory validation.

### Static deflection

**Figure 5:**
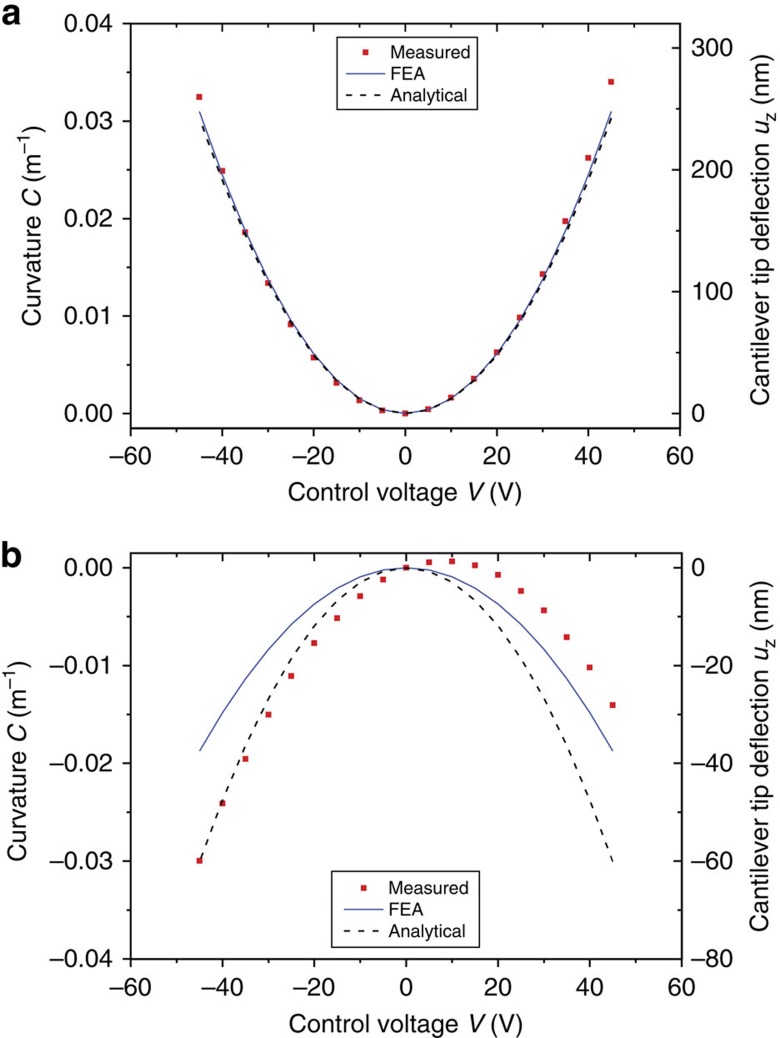
Characteristic V-like and Λ-like NED actuator deflection curves. The active change in curvature and the cantilever tip deflection are shown to depend on the static control voltage. The electrode separation *t*_g_ was designed to be 200 nm. (**a**) Sample of V-like NED actuator, where the cantilever measures *l*_a_=4,000 μm in length, *w*_b_=60 μm in width and has a thickness of *t*_b_=26.5 μm. (**b**) Sample of Λ-like NED actuator, where the cantilever measures *l*_a_=2,000 μm in length, *w*_b_=62 μm in width and has a thickness of *t*_b_=29 μm. (**a**,**b**) Experimental measurements (red dots) and electrostatic structural coupled FEA calculated values (blue solid lines, see Supplementary Figs 4 and 10 for FEA model geometry). The dashed line corresponds to values derived from the analytical model using the equivalent structural angles of −32° for V-shaped and 19° for Λ-shaped NED actuators. The standard deviation for the measurements with the digital holographic microscope was below 1 nm, and is, therefore, not plotted as error bars in either graph.

The test sample preparation for the electromechanical characterization utilizing digital holographic microscopy is given in the Methods section. Measurements of the NED cantilever tip deflections at different control voltages were performed. The active change in curvature was then determined (Fig. 5). Figure 5a shows a typical characteristic actuator deflection curve of a V-like NED actuator sample with the bottom electrode set to high potential. The deflection is an upward movement of the cantilevers free end, which increases quadratically with the control voltage. By arranging the elementary V-like actuators cells on top of the cantilever, the resulting curvature is just as positive. The positive actuation direction can be justified with the top electrode effective structural angle of −32°, which conforms to the theoretical model presented in Fig. 2. Maximum curvature values of 0.034 m^−1^ and deflections of 272 nm were obtained with control voltages of 45 V. As depicted in Fig. 5a, the measured curvatures agree well with the analytical model for *α*=−32°, as well as with the results from the electrostatic structural coupled FEA of the adjusted actuator geometry (Supplementary Fig. 4). The maximum deviation was ≈9 % with the active change in curvature being underestimated in both the simulation and analytical model.

Figure 5b displays the characteristic actuator deflection curve of a Λ-like NED actuator sample, which has an opposite movement to the cantilever's free end compared with the fabricated V-like actuators. The positive and smaller effective structural angle of 19° results in a downward movement in tip deflection, which affirms the prediction of the theoretical model (Fig. 2). This confirms the fundamental influence of the elementary actuator cell topography on the mechanical response of the NED actuator due to electrical excitation. The asymmetry of the quadratic curvature-voltage characteristic in Fig. 5b can be described with a voltage shift of ≈5 V and is also present, although significantly smaller, within the V-like NED actuator sample (Fig. 5a). Further investigations showed that this voltage shift is caused by permanently trapped charges in the alumina insulating layer, which are potentially induced by defects resulting from reactive ion etch (RIE) processes.

## Discussion

The fundamental working principle of the novel electrostatic bending actuator, the theoretical as well as the electrostatic mechanical coupled finite element model, were validated on non-optimized, manufactured and characterized test samples. Therefore, the question arises of how the NED scaling behaviour and optimized parameter combinations can provide much larger cantilever tip deflections and enable actuator deflections widely beyond the initial electrode separation. According to bimorph actuators theory, the cantilever tip deflection scales quadratically with length *l*_a_ of the cantilever^16^,^18^, and the curvature and, therefore, the tip deflection scales in an inversely quadratic relationship with the total thickness of a cantilever^18^ — in simplified case where one layer is much thinner than the other. Obviously, a long and thin cantilever leads to large actuations. Furthermore, it is well known that the ratio of layer thicknesses and the ratio of the elastic moduli are the dominant parameters to achieve high curvatures in bimorph actuators^16^. These trends are similar for the NED actuator; where the cantilever and the top electrode can be assumed as a first and a second layer in a bimorph configuration. However, the various strong transformations of electrostatic forces into lateral forces at different effective structural angles of the top electrode (Fig. 2), makes these trends significantly more complex (Supplementary Fig. 5). To achieve high actuator deflection, the ratio of the cantilever thickness to the top electrode thickness *t*_b_/*t*_e_ and the effective structural angle *α* need to be optimized for a given set of materials with defined moduli of elasticity (Supplementary Fig. 5).

Another important factor in NED cantilever deflection is the electrode separation *t*_g_ and the actuator control voltage *V*. The electrostatic forces and, therefore, the active change in NED curvature, scale with 
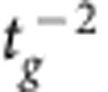
 and *V*^2^ for the case of small top electrode deflections (Supplementary Equation (1)). Thus, smallest possible electrode gaps and high control voltages are theoretically favourable. However, those two parameters are limited by the top electrode pull-in instability, which itself will be influenced by the stiffness, and therefore the thickness *t*_e_, the length and the elastic modulus of the top electrode. The strong influence of the stiffness of top electrode on the NED deflection can be seen from the difference between the analytical and FEA simulated curvature at roof angles α between −10° and 10° in Fig. 2. The analytical model unrealistically assumes the top electrode has infinite stiffness, therefore has a constant electrode separation *t*_g_ during actuation, and creates the highest NED curvatures and deflections at small roof angles. The curvature and deflection will be much smaller with finite and, therefore, more realistic top electrode stiffness at small absolute values of the roof angles. Thus, for the downward scaling of the electrode separation, it is favourable to use thick and short, that is, stiff, top electrodes in practice. However, a reduction in the top electrode length *l*_b_ will automatically decrease the percentage ratio of electrostatic active area to passive area within a NED cantilever. The passive areas mainly include the spacers with their lengths *l*_s_. It is obvious that high ratios of *l*_b_/(*l*_b_+*l*_s_) are necessary for a high NED effect. An increase in top electrode thickness *t*_e_ will further influence the optimal and previously discussed ratio *t*_b_/*t*_e_. To summarize, all previous described parameters need to be taken into account for the investigation of the electrode separation downward scaling. The strongest scaling can be achieved with the most rigid top electrodes. In the case of small top electrode deformations and for ideally stiff top electrodes, the active change in NED curvature scales with 
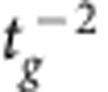
 (Supplementary Equation (21)).

The novel actuator class aims to utilize the huge electrostatic forces at the smallest possible electrode separation. Hence, the limit of the electrode separation downward scaling is of interest. We expect that the smallest electrode gap size is physically restricted by the Casimir effect and possibly, but as yet unknown, technically limited by the sacrificial layer release etch. We expect that the Casimir forces will lead to the sticking of the top to the bottom electrode for very small electrode separations. This effect will thus represent a limit for electrode gap downscaling. Additionally, parasitic effects, such as tunnelling current and increased probability of electrical breakdown at electrode edges, become more prominent at small electrode separation and will influence the actuator performance.

The novel electrostatic actuator approach was demonstrated and validated with MEMS actuators for two different top electrode effective structural angles of −32° and 19° and 200 nm electrode separation. These non-optimized NED actuators reach quasi-static deflection amplitudes up to 272 nm (Fig. 5a) at control voltages of 45 V. This deflection corresponds to a travel range *t*_r_ of 136 % which is already beyond the limit given by the pull-in instability. Assuming feasible NED actuator geometrical values, we predict tens to hundreds of micrometres actuator deflections with electrode separations of 200 nm, cantilever lengths of 2 mm and control voltages of 45 V (Supplementary Fig. 6). This demonstrates that travel ranges above 5,000 % are feasible (Supplementary Fig. 7), but also demonstrate the huge optimization potential for further increase of the travel range widely beyond the electrode separation.

In conclusion, we have presented a novel electrostatic actuator principle that allows deflection amplitudes that are significantly higher than the electrode separation. With this actuator concept high quasi-static as well as high resonant actuation amplitudes are possible at low voltages. We demonstrated NED actuator geometries for positive and negative cantilever curvatures, and, therefore, for upward and downward actuator deflections. A combination of upward with downward deflectable NED actuators permits bidirectional electrostatic actuation, S-shaped cantilever deformation profiles and thus pure out-of-plane tip deflections. The novel actuator class will benefit from the low-power consumption of electrostatic actuators, the simple fabrication process and the integration with CMOS technologies. It further demonstrates a solution to prevent or reduce the occurrence of pull-in related failure mechanisms of MEMS actuators and sensors.

Our primary vision is to investigate MEMS actuators with the smallest possible electrode separation and evaluate the downward scaling limitation of the novel actuator class, given by tunnelling, Casimir or other parasitic effects. A secondary vision is to investigate the possibility to substitute or supplement electroactive actuation materials, and enable electrostatic actuation for bimorph type actuators. The future development of the novel actuator class has to provide deflection and efficiency optimized actuator geometries and manufacturing technologies. In addition to the improvements named of the simple NED actuator geometries presented, further developments have to consider multi-stacked top electrodes for increases in NED effect, bidirectional actuation, S-shaped deformable cantilevers, an investigation of technologies for in-plane and surface micro-machined NED actuators.

## Methods

### Finite element analysis

To investigate and optimize the behaviour of the NED actuator, an FEA model has been developed using the Mechanical Ansys Parametric Design Language (MAPDL) module from ANSYS 15.0. For this model, an approximation of the behaviour of a cantilever beam for small deflections was applied. However, the electrodes on the surface of the beam were acting in the domain of large deflection (electrode displacement is larger than a tenth of electrode thickness). For that reason, the model was solved as a non-linear model. Moreover, the model was solved as a two-dimensional model. As presented in the Results section, the structure is composed of a beam on which a series of actuators are aligned. Each actuator acts independently on the underlying section of beam. Taking into consideration the periodicity of the actuators along the beam and the vertical symmetry of each actuator, the model geometry was reduced to half a cell. The model used the PLANE223 coupled-field high-order element from MAPDL element library, allowing coupled electrostatic and structural analysis. The different materials, including well-oriented anisotropic silicon, were applied to the model (Supplementary Fig. 4). Following a mesh convergence analysis, the model was meshed and the elements correctly set-up according to the element description, at the electrode/gap interface to allow local transfer of forces from the electrostatic to the structural domain, and local transfer of displacements from the structural to the electrostatic domain. The structural boundary conditions were applied to reproduce the half-cell symmetry and the Euler Bernoulli hypothesis associated with small deflections. This was achieved by the alignment of the nodes at the cell/cell interface, which was imposed by applying an extra element, the MPC184 link element. After solving the model, the post-process step was performed, calculating the curvature of the entire beam 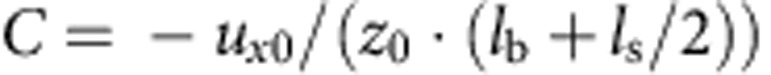 using the *x*-displacement of the bottom point of the cell interface 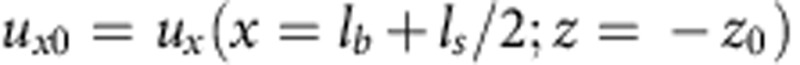, the height of the neutral fibre *z*_0_ and the length of the half cell 
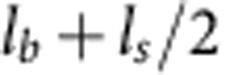
 (Supplementary Fig. 11).

### Sample fabrication and preparation

The fabricated NED actuators consist of two main parts: a silicon cantilever with topography and a layer stack. The latter includes (from the bottom up): insulator, bottom electrode, sacrificial layer and top electrode thin film. The substrates used to fabricate the NED actuators were bonded-silicon-on-insulator wafers (BSOI) with a 30-μm-thick silicon device layer, 1-μm-thick buried silicon dioxide and a 400-μm-thick silicon handle wafer. The thickness of the NED cantilever was defined by the thickness of the device layer minus the depth of the first etches to form the surface topography (Supplementary Fig. 1). The fabrication process is based on bulk micro-machining. The process steps are schematically summarized in Supplementary Fig. 3. To define the NED topography, V-shaped grooves (V-like NED actuator samples, Fig. 2c) or Λ-shaped plateaus bars (Λ-like NED actuator samples, Fig. 2d) are etched into the surface of the device silicon layer via TMAH wet etching and a silicon dioxide hard mask (Supplementary Fig. 3a). Second, a 400-nm amorphous alumina (Al_2_O_3_) insulation layer is deposited via atomic layer deposition (Supplementary Fig. 3b). The purpose of this layer is to act as an etch stop during the RIE of the bottom electrode and to serve as a landing area for the top electrode on the NED cantilever. Al_2_O_3_ was chosen due to the fact that it is highly stable during the hydrogen fluoride (HF) gas phase etching of the sacrificial layer. On top of the alumina layer a 500-nm amorphous TiAl bottom electrode was deposited via magnetron sputter deposition (Supplementary Fig. 3c). Because of the small separation of 200 nm between the top and bottom electrode, the roughness of the bottom electrode is of importance to prevent short-circuits, discharging spikes or tunnelling currents between the top and bottom electrode. Amorphous TiAl has a smaller roughness compared with other metals, such as aluminium (Al) or silicon-coper doped Al (AlSiCu), and is stable during the HF-gas phase release etching. The lateral structuring to define the shape of the TiAl bottom electrode was done by photolithography in combination with RIE with a selective stop on the underlying Al_2_O_3_ layer. Additionally, landing areas within the bottom electrode were defined via lateral structuring to enable the mechanical contact of the top electrode with the underling insulation Al_2_O_3_ layer. Subsequently, the sacrificial layer, 200 nm thin undoped silicon glass, is deposited by plasma enhanced chemical vapour deposition. Thereafter, the sacrificial layer was structured via RIE (Supplementary Fig. 3d). Next, the deposition and structuring of the amorphous TiAl top electrode was achieved analogously to the bottom electrode (Supplementary Fig. 3e). The top electrode was deposited with thicknesses of 500 nm (V-like NED actuator) and 1,000 nm (Λ-like NED actuator), and had a mechanical contact to the alumina insulation layer within the landing areas previously defined within the bottom electrode and the sacrificial layer. Thus, the top and the button electrode were laterally insulated via the alumina insulation layer and vertically insulated to the silicon device layer of the BSOI wafer. To define the vertical thickness of the NED cantilever, the 400-μm-thick handling layer of the BSOI wafer was structured by means of back side lithography and back side anisotropic wet etching in TMAH solution which selectively stopped at the buried oxide layer of the BSOI wafer (Supplementary Fig. 3f). The already processed front side of the substrate was protected from the etching solution with a cover box. The lateral geometry of the NED cantilever (Fig. 4) was defined by high aspect ratio, 10-μm-wide and 30-μm-deep trenches, etched in silicon on the front side of the wafer via deep RIE (Supplementary Fig. 3g). Before the sacrificial layer release etch, the NED test samples were diced by a conventional wafer dicing saw. To protect free standing and fragile NED cantilevers during the dicing process, the front side of the wafer was covered by a photoresist layer. After dicing, the protection photoresist of the samples was stripped on chip level. Finally, the silicon dioxide sacrificial layer release etch was achieved with HF vapour on chip level. This release etch ensured the separation of the electrodes by creating an ≈200-nm-thin conformal air gap, and simultaneously removing the buried oxide layer from the cantilever reverse side (Supplementary Fig. 3h).

The released silicon chips, measuring ≈6 × 4 mm, contain four different NED actuators with specific layout dimensions. To perform the electromechanical characterization, the single chips were glued in dual-inline packages with a conductive silver glue and wire bonded on TiAl bond pads. It was ensured that the highly doped device layer and the less doped handle layer of the BSOI wafers were in contact with the conductive glue. This was accomplished by pulling the glue droplet along the full height of the test chip. Utilizing wedge-bonding each electrode, as well as the cantilever, were electrically connected to individual pins of the package. The electromechanical measurements were performed by mounting the packaged test chips in zero-force-sockets, which were connected through a self-designed manual switching matrix. By plugging electric jumpers of the switching matrix, each electrode of a single actuator could be addressed individually. The switching matrix was connected to the power supply by a coaxial cable.

### Deflection measurement set-up

For the electromechanical characterization of the cantilever tip deflection, the bottom electrode was set to high potential, controlled by a digital voltage supply. The top electrode, silicon handle and device layer were grounded during this test configuration. The out-of-plane NED actuator deflection *u*_z_ was measured with a commercial digital holographic microscope at the free end of the cantilever. The digital holographic microscopy^20^ utilizes a video camera to record a hologram generated by the interference of laser light between a reference wave and the reflected wave front from the object to be analysed. Numerical procedures were then applied to reconstruct a three-dimensional image with sub-nanometre vertical resolution. The NED bottom electrode control voltage *V* was increased stepwise from −45 to 45 V in steps of 5 V. At each step the deflection was measured 10 times, by switching the voltage source off and on to ensure reproducible measurements. The active change in cantilever curvature *C* was calculated with 
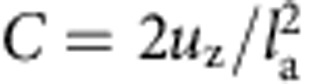
 and was plotted versus the voltage values (Fig. 5). Utilizing curvature instead of displacement values allows the comparison of different NED actuator types with or at various cantilever lengths. Further the measured results can be directly compared with theoretical values from the FEA and the analytical model.

## Additional information

**How to cite this article:** Conrad, H. *et al.* A small-gap electrostatic micro-actuator for large deflections. *Nat. Commun.* 6:10078 doi: 10.1038/ncomms10078 (2015).

## Supplementary Material

Supplementary Information Supplementary Figures 1-13 and Supplementary Methods
