# Supplementary material for: A small-gap electrostatic micro-actuator for large deflections
Source: Nat Commun. 2015 Dec 11;6:10078. doi: 10.1038/ncomms10078 (PMC4682043; doi:10.1038/ncomms10078)
Supplement: Supplementary Information — Supplementary Figures 1-13 and Supplementary Methods [file ncomms10078-s1.pdf]

Supplementary Figures

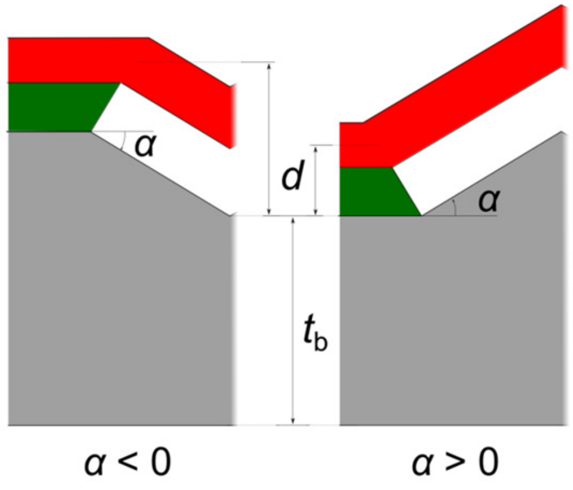

Supplementary Figure 1 | Schematic presenting the evolution of the geometry of the structure as a function of the roof angle α as well as the definition of the beam thickness t_b and the equivalent lever length d.

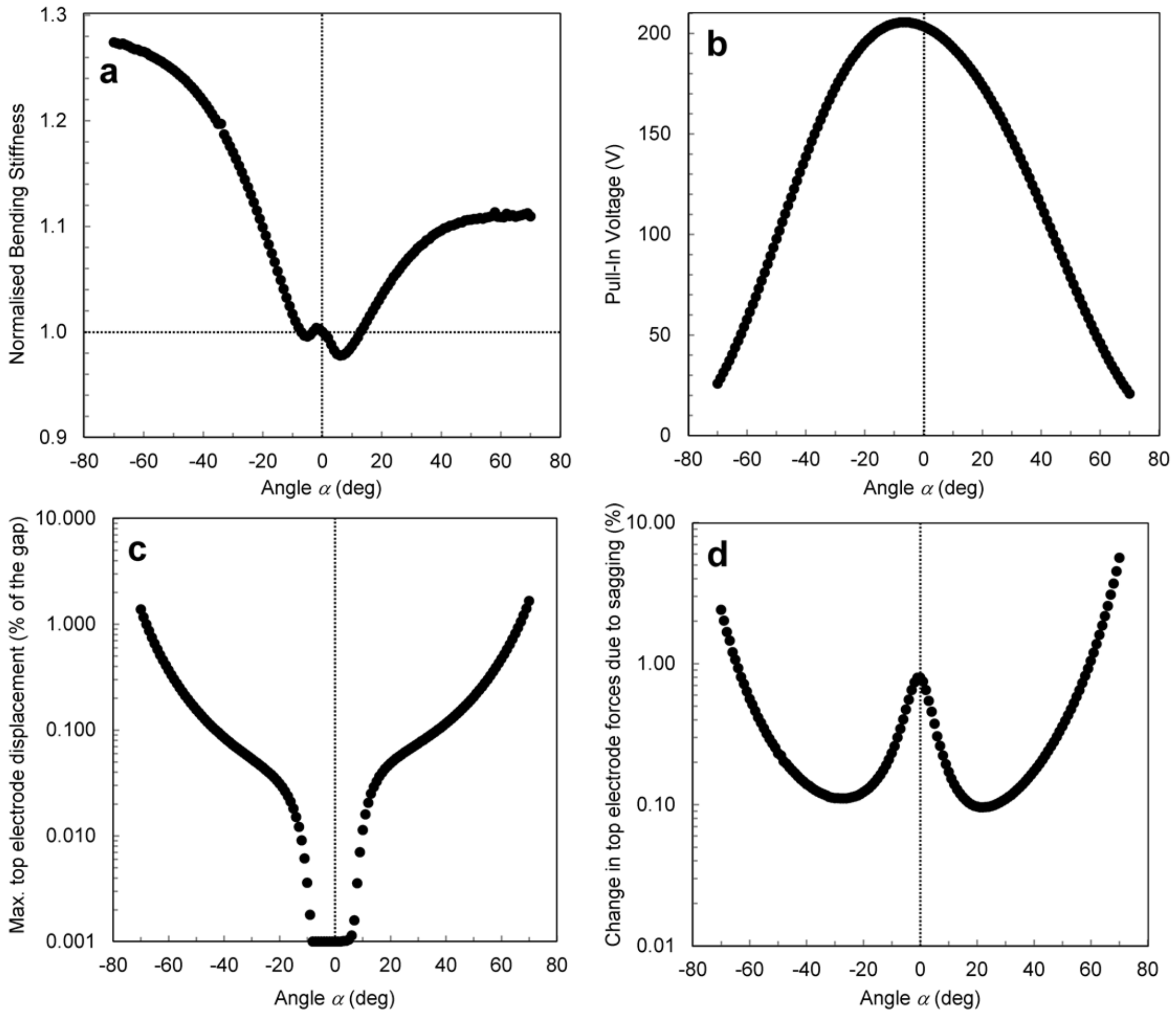

Supplementary Figure 2 | Additional values of the FEA model presented in Fig. 2 and 3. The calculation was done with structural FEA (**a**) or electrostatic mechanical coupled FEA (**b-d**) and with the following set of parameters: l_b = 2.5 µm, t_g = 100 nm, t_b = 6 µm, t_e = 200 nm, l_s = 0.5 µm, E = 169 GPa for both electrodes and E = 266 GPa for the spacers. (**a**) Graph presenting the bending stiffness of the Λ- and V-shaped NED actuators, normalised to the bending stiffness of the beam defined for α = 0. (**b**) The top electrode pull-in voltage depending on the roof angle α. (**c**) The maximum top electrode sag displacement, in percent of the electrode separation, at electrostatic control voltages of 10 V. (**d**) The influence of the top electrode sagging to the generated electrostatic forces at control voltages of 10 V. The electrostatic forces were calculated with deflected and with fixed top electrodes (u_x = u_y = 0 at the air – top electrode interface). The relative increase of electrode electrostatic forces due to the sagging of the top electrode is given as a function of the roof angle α.

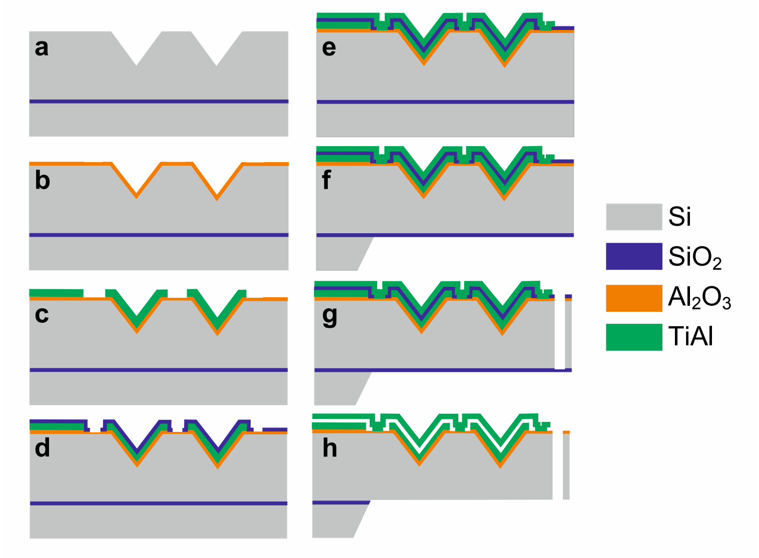

Supplementary Figure 3 | schematic presentation of the NED technology process flow. (**a**) The process starts with TMAH etching of V-shaped groves at the surface of the BSOI device layer. (**b**) Atomic layer deposition of 400 nm alumina thin film as an insulation layer. (**c**) Sputtering deposition and lateral RIE structuring of 500 nm titanium aluminide thin film as a lower electrode. (**d**) Chemical vapour deposition and lateral RIE structuring of 200 nm silicon dioxide thin film as a sacrificial layer. (**e**) Sputtering deposition and lateral RIE structuring of 500 nm (V-like NED actuators) or 1000 nm (Λ-like NED actuator) titanium aluminide thin film as an upper electrode. (**f**) Bottom side TMAH etching of groves within the handling layer of the BSOI wafer. (**g**) Deep reactive ion etching of trenches to laterally define the cantilever edge. (**h**) Hydrogen fluoride vapour etching of the silicon dioxide sacrificial layer along with the BSOI wafer buried oxide to finally release the elementary actuator cells as well as cantilever.

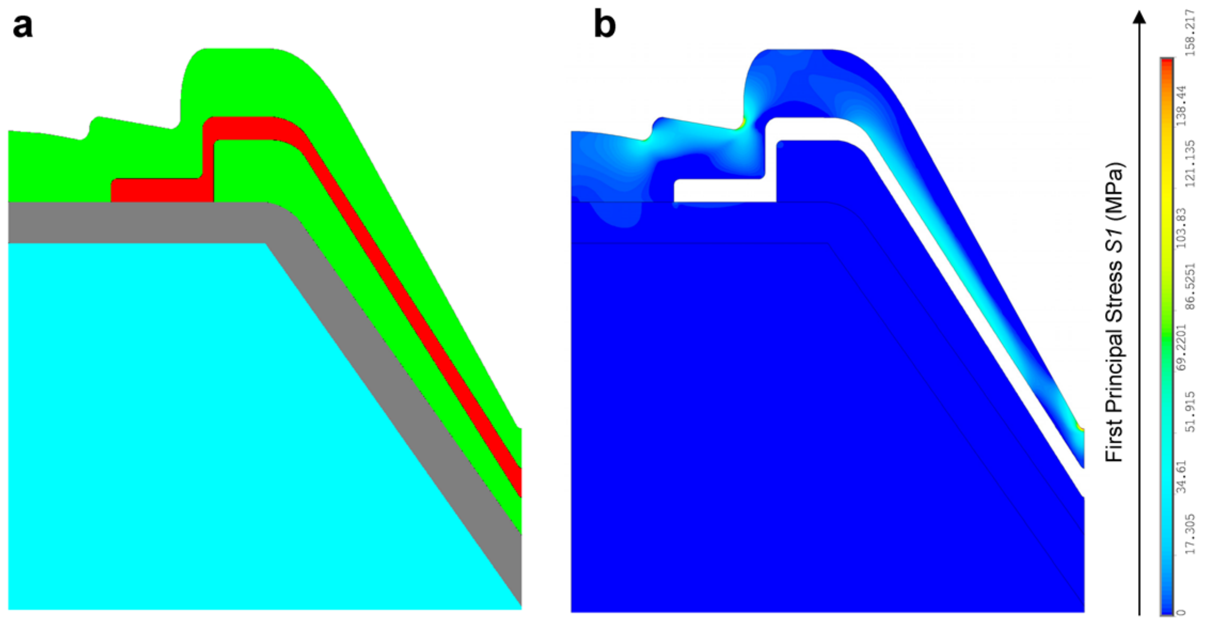

Supplementary Figure 4 | Detail of the elementary actuator cell geometry for the FEA of V-like NED actuator. (**a**) The structural domain consists of the single crystalline silicon cantilever (coloured cyan), the alumina insulation layer (coloured grey), the bottom and the top electrodes (coloured green). The electrostatic domain is represented by the air gap (coloured red). Both domains are coupled at their interfaces. For the FEA model the following material parameters were used: silicon with anisotropic material constants $E_x = E_z = 169$ GPa, $E_y = 130$ GPa, $G_{xy} = G_{yz} = 79.4$ GPa, $G_{xz} = 50.9$ GPa, $v_{xy} = 0.28$, $v_{xz} = 0.064$, $v_{yz} = 0.36$; alumina pure isotropic $E = 266$ GPa, $v = 0.25$; titanium aluminide pure isotropic $E = 140$ GPa, $v = 0.25$. (**b**) Contour plot of the first principal stress *S1*, calculated for an applied electrostatic control voltage of 45 V.

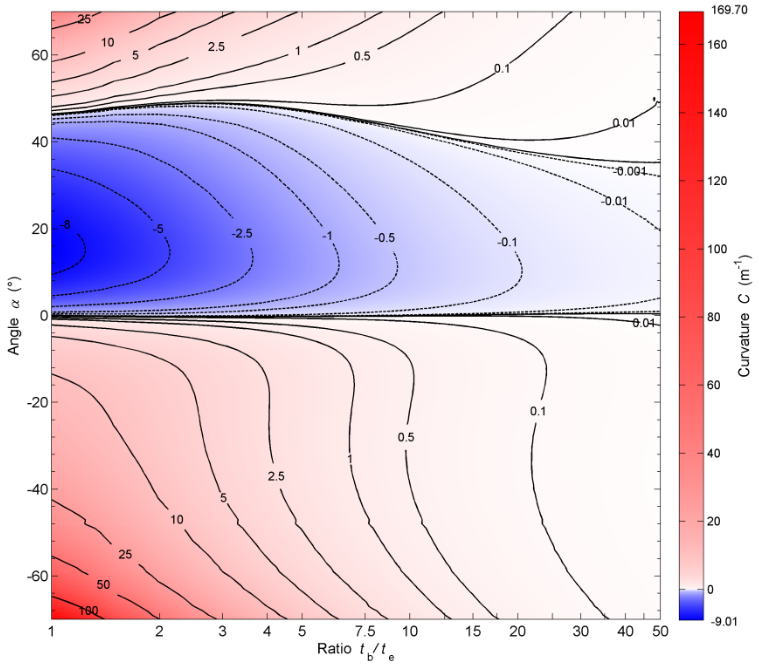

Supplementary Figure 5 | The controlled change in curvature *C* for V- and Λ-shaped NED actuators as a function of the ratio of beam thickness t_b to the top electrode thickness t_e, and as a function of the elementary actuator roof angle α. Values are calculated with electrostatic mechanical coupled finite element analysis for the following set of parameters: t_g = 200 nm, control voltage V = 45 V, l_b = 10 μm, l_s = 1 μm, and t_e = 1 μm. The cantilever and the top electrode are assumed to be made of silicon with E = 169 GPa and the ratio of their elastic moduli equals 1. The spacer was assumed to be made of alumina with E = 266 GPa. There are no collapsed top electrodes for any set of parameters. Thus, the top electrode pull-in instability voltage is higher than 45 V for all simulated geometries.

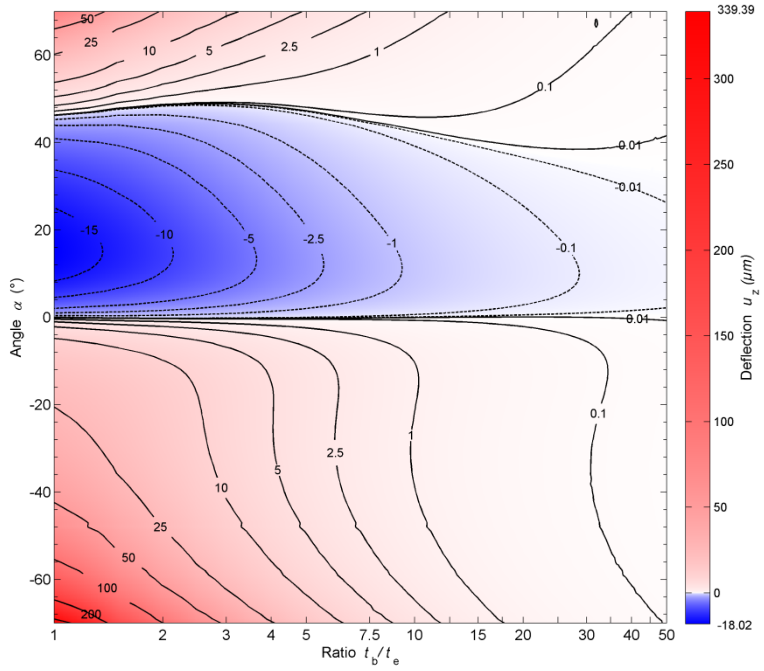

Supplementary Figure 6 | The V- and Λ-shaped NED cantilever tip deflection u_z as a function of the ratio of beam thickness t_b to the top electrode thickness t_e and as a function of the elementary actuator roof angle α. Values are calculated from the cantilever curvatures, given in Supplementary Fig. 5 for a l_a = 2 mm long NED cantilever.

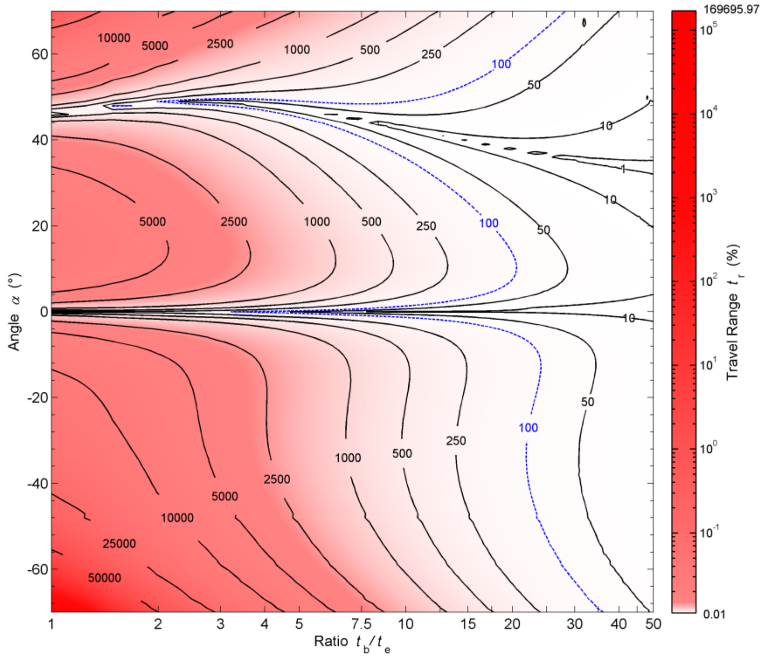

Supplementary Figure 7 | The V- and Λ-shaped NED travel range t_r as a function of the ratio of beam thickness t_b to the top electrode thickness t_e, and as a function of the elementary actuator roof angle α. Values are calculated from the absolute value of tip deflection for a l_a = 2 mm long cantilever, as given in Supplementary Fig. 6, and an electrode separation t_g of 200 nm. The top electrode pull-in instabilities were not reached at U = 45 V, therefore the maximum achievable travel ranges can be much higher for each single data point.

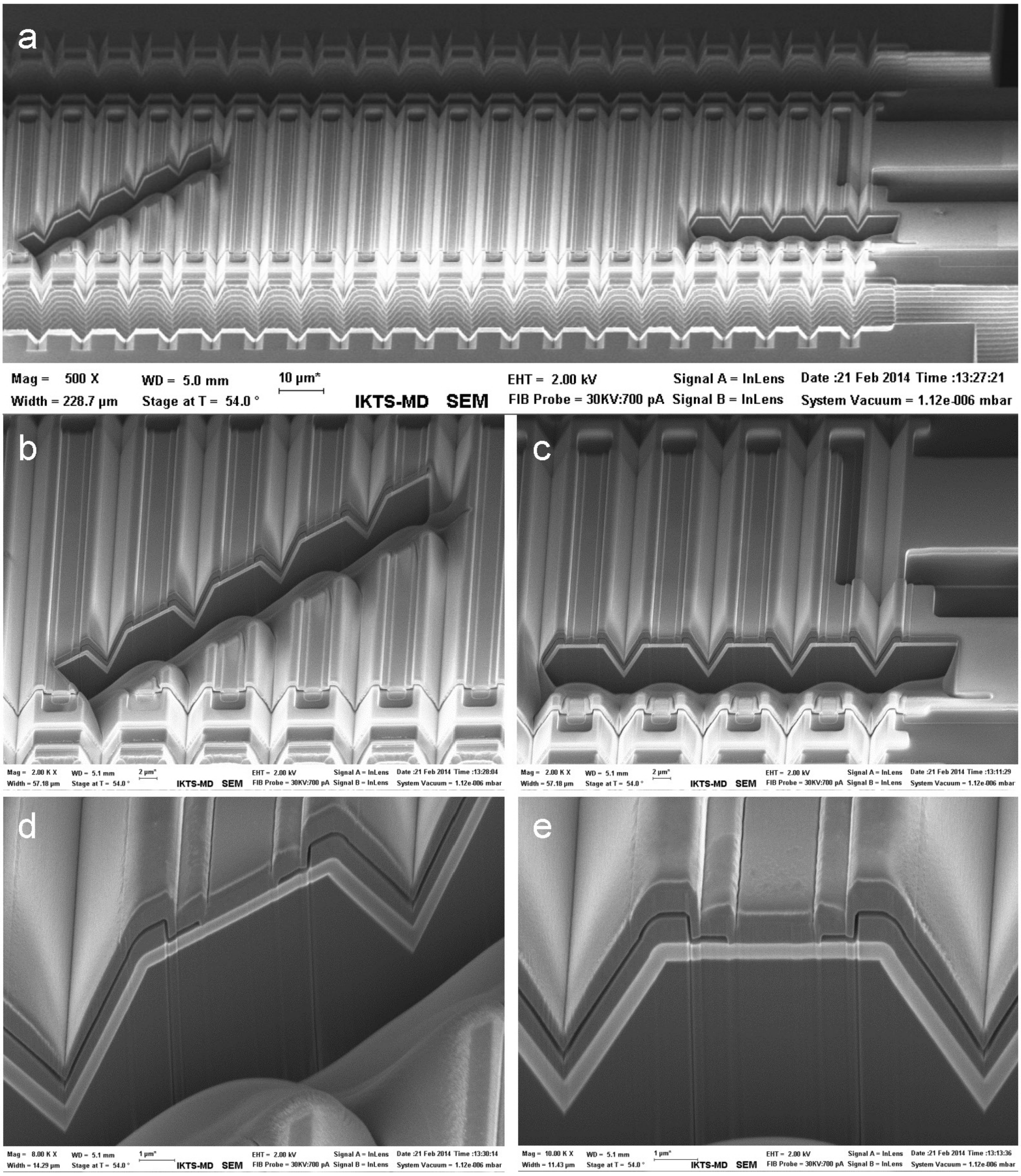

Supplementary Figure 8 | Scanning electron micrograph of a sample of V-like NED actuator. (**a**) Two cuts are made with a focused ion beam after sacrificial layer releases etch. (**b**-**e**) Cross sections that detail the NED elementary actuator cell.

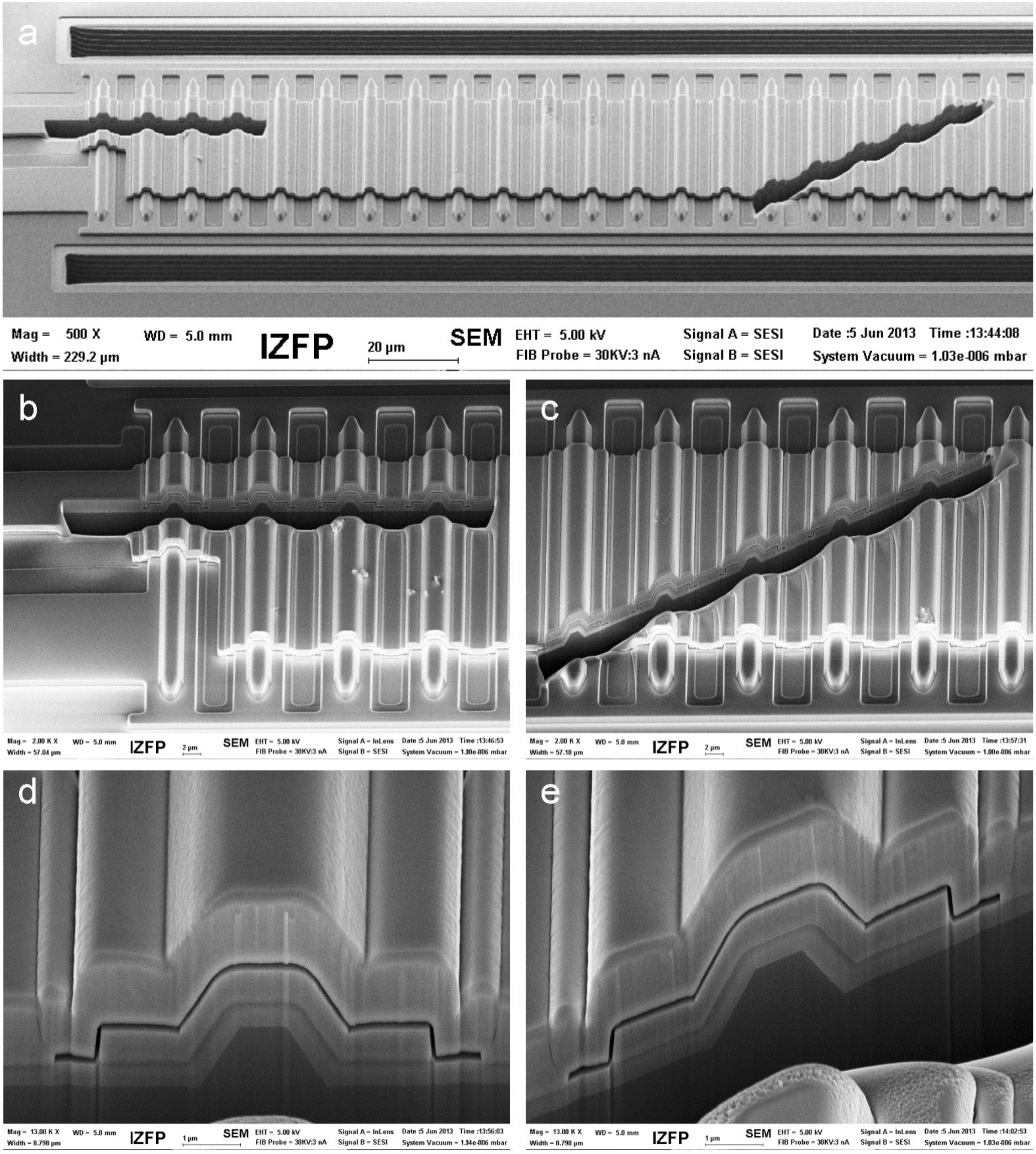

Supplementary Figure 9 | Scanning electron micrograph of a sample of Λ-like NED actuator. (**a**) Two cuts are made with a focused ion beam after sacrificial layer releases etch. (**b-e**) Cross sections that detail the NED elementary actuator cell.

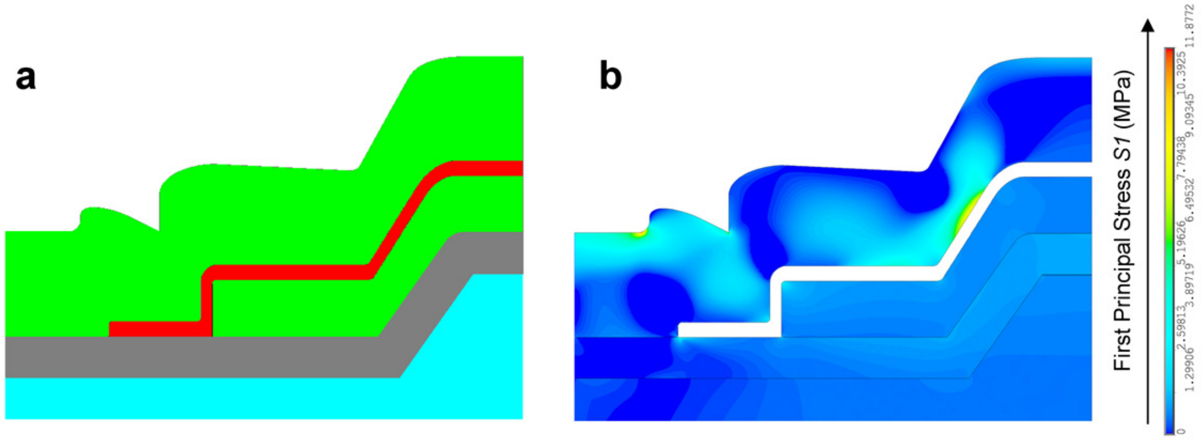

Supplementary Figure 10 | Detail of the elementary actuator cell geometry for the FEA of Λ-like NED actuator. (**a**) The structural domain consists of the single crystalline silicon cantilever (coloured cyan), the alumina insulation layer (coloured grey), the bottom and the top electrodes (coloured green). The electrostatic domain is represented by the air gap (coloured red). Both domains are coupled at their interfaces. The same material parameters as the FEA model of the V-like NED actuator (Supplementary Fig. 4) were used. (**b**) Contour plot of the first principal stress *S1*, calculated for an applied electrostatic control voltage of 45 V.

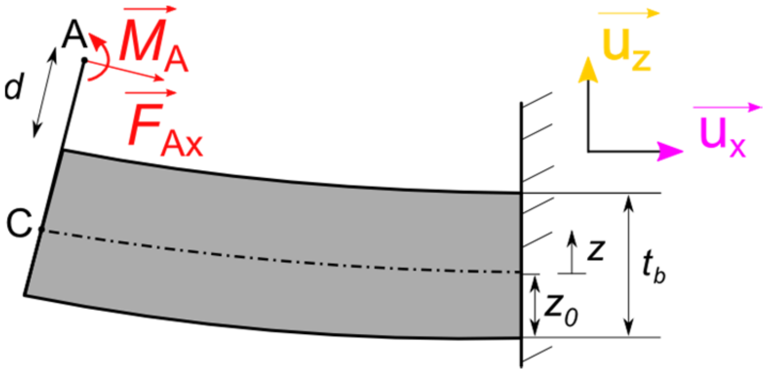

Supplementary Figure 11 | Schematic presenting the single side clamped beam, thickness t_b and young modulus E, the lever of length d at its end, the reactions F_{Ax} and M_A applied at the end of the lever and the position of the neutral fibre at a distance z_0 from the bottom fibre of the beam.

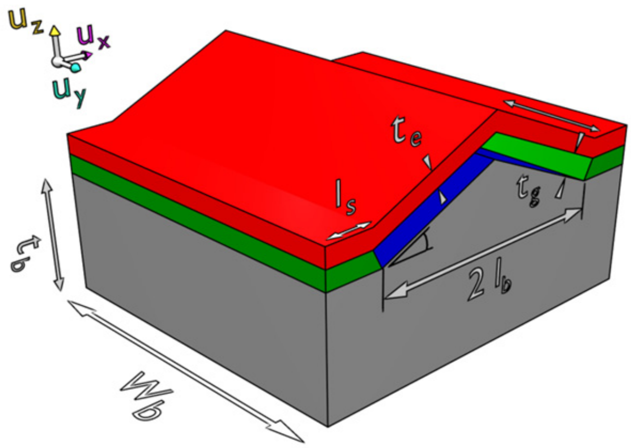

Supplementary Figure 12 | Schematic presenting a roof shaped elementary actuator cell. The base element of the actuator beam, and the different parameters used for the development of the analytical model: 2 l_b the length of the cell, w_b the width of the cell, α the angle from $\overrightarrow{u_x}$ to the roof slope, t_g the thickness of the air gap or the electrode separation, t_e thickness of the top electrode, l_s the length of the spacer (coloured in green) and t_b the thickness of the continuous beam under the roof-shaped structure.

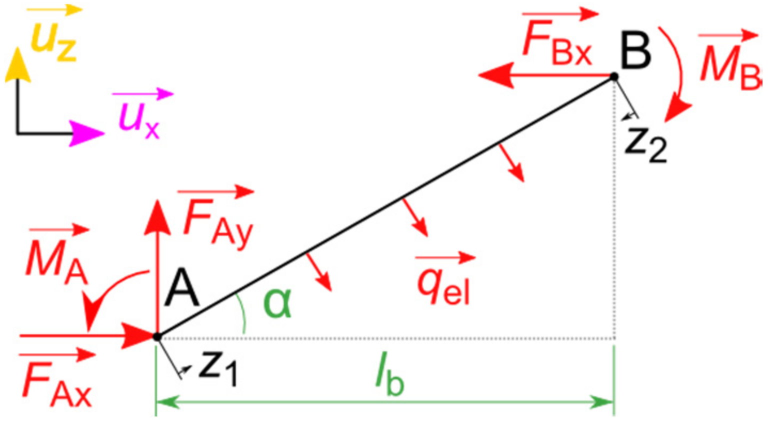

Supplementary Figure 13 | Schematic presenting the reactions F_{Ax}, F_{Ay}, F_{Bx} and M_A due to the electrostatic pressure q_{el} applied to half of the top electrode. l_b being the half length of the actuator cell.

Supplementary Methods

Analytical model for the NED actuators

A NED actuator consists of many actuator cells in series that are expected to operate independently and identically. For this reason, the behaviour of the whole NED actuator can be described by modelling one actuator cell. Furthermore, due to the x-symmetry only half of an actuator cell with the length l_b (Supplementary Fig. 12) needs to be considered.

The following development is associated with a 2D structure with V- or Λ-shaped geometry of the top electrode. The width of the model is assumed to be equal to w_b (Supplementary Fig. 12). The cross-section can be approximated as shown in Supplementary Fig. 13. In this approximation, and for simplicity, we neglect the spacer length $l_s << l_b$ and assume that both electrodes are made of the same material, i.e. they have the same Young's modulus E.

Reaction forces and moments of the top electrode

The forces generated in the electrode gap cause the electrostatic bending of the NED actuator. Assuming negligible changes of the electrode separation t_g (see Supplementary Fig. 2c and 2d), i.e. small top electrode displacements, the distributed load q_{el} can be calculated as follows:

$$q_{\mathrm{el}} = \frac{1}{2}\varepsilon_0\varepsilon_{\mathrm{r}}\frac{V^2}{t_{\mathrm{g}}^2}w_{\mathrm{b}} \tag{1}$$

Here, ε_0 is the permittivity of a vacuum and ε_r is the relative permittivity of the fluid filling the gap. The fluid is set to be ordinary air with ε_r = 1.

In Supplementary Fig. 13 the top electrode between the bearing points A and B is approximated as an ideal stiff rod. The electrostatic pressure acts on this rod and creates the five reactions which are F_{Ax}, F_{Ay}, M_A, F_{Bx} and M_B in A and B respectively. To solve for these five reactions in a linear equation system, the same number of equations are necessary. Three of them are composed of the fundamental balance of forces and momentums:

$$0 = F_{\mathrm{Ax}} - F_{\mathrm{Bx}} - q_{\mathrm{el}}l_{\mathrm{b}}w_{\mathrm{b}}\tan(\alpha) \tag{2}$$

$$0 = F_{\mathrm{Ay}} - q_{\mathrm{el}}l_{\mathrm{b}}w_{\mathrm{b}} \tag{3}$$

$$0 = -M_{\mathrm{A}} + M_{\mathrm{B}} - F_{\mathrm{Bx}}l_{\mathrm{b}}w_{\mathrm{b}}\tan(\alpha) + \frac{q_{\mathrm{el}}w_{\mathrm{b}}{l_{\mathrm{b}}}^2}{2\cos(\alpha)^2} \tag{4}$$

In these equations q_{el} is the distributed load created by the electrostatic forces as mentioned previously. The angle α describes the slope between the top electrode and the x-axis. Two additional equations can be gained by applying the second theorem of Castigliano. It states that the work W performed by indeterminate reactions, in our case F_{Bx} and M_B, is zero. This can be used to solve for the indeterminate reactions. We assume that the mechanical work is performed only because of bending. So one can write:

$$W = \sum_i \frac{1}{2}\int_0^{\mathrm{l}_i}\frac{M_{\mathrm{b}i}^2}{K_i}dz_i = 0 \tag{5}$$

The parameter K_i describes the bending stiffness of the element which is bent around the Y-axis with the corresponding moment M_{bi}. For a rectangular cross section (YZ-plane) of thickness t and width w_b it can be calculated as follows:

$$K = E\frac{w_\mathrm{b}\,t^3}{12} \tag{6}$$

From equation (5) the displacement v and inclination φ resulting from the indeterminate force and the momentum can be written as:

$$v_\mathrm{Fbx} = \sum_i \int_0^{l_i} \frac{M_{\mathrm{b}i}}{K_i}\frac{\partial M_{\mathrm{b}i}}{\partial F_\mathrm{Bx}} dz_i = 0 \tag{7}$$

$$\varphi_\mathrm{MB} = \sum_i \int_0^{l_i} \frac{M_{\mathrm{b}i}}{K_i}\frac{\partial M_{\mathrm{b}i}}{\partial M_\mathrm{B}} dz_i = 0 \tag{8}$$

The two evolving bending moments M_{bi} are formulated for the top electrode according to Supplementary Fig. 12 as follows:

$$M_{b1} = F_{Ay}z_1\cos(\alpha) - F_{Ax}z_1\sin(\alpha) - \frac{1}{2}q_{el}z_1^2 - M_A \quad with \quad 0 \le z_1 \le \frac{l_\mathrm{b}}{4\cos(\alpha)} \tag{9}$$

$$M_{b2} = F_{Bx}z_2\sin(\alpha) - \frac{1}{2}q_{el}z_2^2 - M_B \quad with \quad 0 \le z_2 \le \frac{l_\mathrm{b}}{4\cos(\alpha)} \tag{10}$$

Substituting equations (2), (3) and (4) into (9) and (10), and with the use of the two additional equations (7) and (8), the five reactions can then be solved as a linear equation system. The results are:

$$F_\mathrm{Ax} = q_\mathrm{el}w_\mathrm{b}l_\mathrm{b}\frac{1}{2\tan(2\alpha)} \tag{11}$$

$$F_\mathrm{Ay} = q_\mathrm{el}w_\mathrm{b}l_\mathrm{b} \tag{12}$$

$$M_\mathrm{A} = M_\mathrm{B} = q_\mathrm{el}w_\mathrm{b}l_\mathrm{b}\frac{l_\mathrm{b}}{12\cos(\alpha)^2} \tag{13}$$

$$F_\mathrm{Bx} = q_\mathrm{el}w_\mathrm{b}l_\mathrm{b}\frac{\sin(3\alpha)+\sin(\alpha)}{8\cos(\alpha)^3\sin(\alpha)^2} \tag{14}$$

The bending of the actuator cell

The top electrode is mounted to the substrate in bearing point A. For that reason the reactions in A tend to deform the substrate as illustrated in Supplementary Fig. 11. We assume a constant substrate thickness t_b for any roof angle α and define this thickness as illustrated in Supplementary Fig. 1. It can be seen that the reactions of the top electrode will be transferred over an additional lever through the substrate. The definition of substrate thickness for positive and negative roof angles implies that two cases of lever length d have to be considered:

$$d = \begin{cases} -l_\mathrm{b}\tan(\alpha) + \left(t_\mathrm{g} + \frac{1}{2}t_\mathrm{e}\right)\cos(\alpha), & \alpha < 0 \\ \left(t_\mathrm{g} + \frac{1}{2}t_\mathrm{e}\right)\cos(\alpha), & \alpha > 0 \end{cases}, \tag{15}$$

where t_e denotes the thickness of the top electrode. For $\alpha < 0$ the electrostatic forces will be indirectly transferred from the top electrode to the rest of the cantilever by an ‘overhanging part’ of the top electrode. The sum of the forces F_{Ax} and F_{Ay} creates an angle of 2α with the top electrode. The

projection of its component along the top electrode axis and a second projection along the $\overrightarrow{u_x}$ direction allows the expression of the force acting on the cantilever. For $\alpha > 0$, the electrostatic forces will be directly transformed from the top electrode to the rest of the structure. Consequently, two cases apply for the force F_{Ax}:

$$F_{Ax} = \begin{cases} q_{\mathrm{el}} w_{\mathrm{b}} l_{\mathrm{b}} \dfrac{1}{4\tan(\alpha)}, & \alpha < 0 \\ q_{\mathrm{el}} w_{\mathrm{b}} l_{\mathrm{b}} \dfrac{1}{2\tan(2\alpha)}, & \alpha > 0 \end{cases} \tag{16}$$

The deformation of the substrate is a superposition of tension or compression and bending. Therefore, two associated equilibria have to be fulfilled:

$$0 = F_{\mathrm{Ax}} + \int_{-z_0}^{t_{\mathrm{b}}-z_0} \varepsilon\, E\, w_{\mathrm{b}} dz \tag{17}$$

$$0 = F_{\mathrm{Ax}}(d + t_{\mathrm{b}} - z_0) + \int_{-z_0}^{t_{\mathrm{b}}-z_0} \varepsilon\, E\, w_{\mathrm{b}}\, z\, dz - M_{\mathrm{A}} \tag{18}$$

In these equations ε is the strain, which is distributed along the interface between the elementary actuator cells (YZ-plane). From basic beam theory this can be expressed simply as a product of the curvature *C* and the height *z* along the interface in Z-direction:

$$\varepsilon = -C\, z \tag{19}$$

The integrals in equation (17) and (18) utilise z_0, which is the height of the neutral fibre in a beam, thus summing at point C that is defined in Supplementary Fig. 11. Solving both equations leads to the following expression for the curvature:

$$C = -\frac{6(F_{Ax}(2d + t_b) + 2M_A)}{E\, w_b\, {t_b}^3} \tag{20}$$

Substituting all implicit dependencies gives the curvature in explicit notation for both cases:

$$C = \begin{cases} \dfrac{l_b V^2 \varepsilon_0 \left(3t_b \cos(\alpha)^2 + 3t_e \cos(\alpha)^3 + 6\, t_g \cos(\alpha)^3 - l_b \tan(\alpha) - 6\, l_b \cos(\alpha)^2 \tan(\alpha)\right)}{-2\, E\, t_b^3\, t_g^2 \cos(\alpha)^2 \tan(\alpha)}, \alpha < 0 \\ \dfrac{l_b V^2 \varepsilon_0 \left(6t_b \cos(\alpha)^2 + 6t_e \cos(\alpha)^3 + 12\, t_g \cos(\alpha)^3 - l_b \tan(2\alpha)\right)}{-2\, E\, t_b^3\, t_g^2 \cos(\alpha)^2 \tan(2\alpha)}, \quad \alpha > 0 \end{cases} \tag{21}$$

In these equations (21) the modulation of the bending stiffness *K* of the substrate for different roof angles α is neglected. As demonstrated in Supplementary Fig. 2a, the maximum deviation is 11 % and 28 % for positive and negative angles respectively in the given range of $-70° \leq \alpha \leq 70°$ and for the given set of parameters.
